# The M3 phosphorylation motif has been functionally conserved for intracellular trafficking of long-looped PIN-FORMEDs in the Arabidopsis root hair cell

**DOI:** 10.1186/1471-2229-13-189

**Published:** 2013-11-26

**Authors:** Daisuke Sasayama, Anindya Ganguly, Minho Park, Hyung-Taeg Cho

**Affiliations:** 1 Department of Biological Sciences and Plant Genomics and Breeding Institute, Seoul National University, Seoul 151-742, Korea; 2 Organization of Advanced Science and Technology, Kobe University, 1-1 Rokkodai-cho, Nada-ku, Kobe, Hyogo 657-8501, Japan; 3 Current address, Department of Biology, Washington University, 1 Brookings Drive, Saint Louis, MO 63130, USA

**Keywords:** Auxin, Auxin transport, Hydrophilic loop (of PINs), Phosphorylation, PIN-FORMED (PIN), Protein trafficking, Root hair

## Abstract

**Background:**

PIN-FORMED (PIN) efflux carriers contribute to polar auxin transport and plant development by exhibiting dynamic and diverse asymmetrical localization patterns in the plasma membrane (PM). Phosphorylation of the central hydrophilic loop (HL) of PINs has been implicated in the regulation of PIN trafficking. Recently, we reported that a phosphorylatable motif (M3) in the PIN3-HL is necessary for the polarity, intracellular trafficking, and biological functions of PIN3. In this study, using the root hair system for PIN activity assay, we investigated whether this motif has been functionally conserved among long-HL PINs.

**Results:**

Root hair-specific overexpression of wild-type PIN1, 2, or 7 greatly inhibited root hair growth by depleting auxin levels in the root hair cell, whereas overexpression of M3 phosphorylation-defective PIN mutants failed to inhibit root hair growth. Consistent with this root hair phenotype, the PM localization of M3 phosphorylation-defective PIN1 and PIN7 was partially disrupted, resulting in less auxin efflux and restoration of root hair growth. Partial formation of brefeldin A-compartments in these phosphorylation-mutant PIN lines also suggested that their PM targeting was partially disrupted. On the other hand, compared with the PIN1 and PIN7 mutant proteins, M3-phosphorylation-defective PIN2 proteins were almost undetectable. However, the mutant PIN2 protein levels were restored by wortmannin treatment almost to the wild-type PIN2 level, indicating that the M3 motif of PIN2, unlike that of other PINs, is implicated in PIN2 trafficking to the vacuolar lytic pathway.

**Conclusions:**

These results suggest that the M3 phosphorylation motif has been functionally conserved to modulate the intracellular trafficking of long-HL PINs, but its specific function in trafficking has diverged among PIN members.

## Background

Auxin plays important roles in plant growth and development. Directional cell-to-cell transport and the formation of concentration gradients of auxin are pivotal for its biological function. Diffusive cellular auxin efflux is not effective because of ionization of intracellular auxin, so asymmetrically localized auxin efflux carriers in the plasma membrane (PM) play a critical role in polar auxin transport [1].

PIN-FORMED (PIN) auxin efflux carrier proteins show a distinctive asymmetrical subcellular distribution, which is dynamically regulated by environmental and developmental cues, and their mutations cause defects in auxin gradient formation and plant growth and development [2]. The *Arabidopsis* PIN family consists of eight members, among which six PINs (PIN1–4, 6 and 7) have a long central hydrophilic loop (HL; 298–377 amino acid residues) connecting the five transmembrane (TM) helices on each end. These ‘long’ PINs mainly localize to the PM. Conversely, the other two PINs (PIN5 and 8) have a very short (27–46 residues) HL and localize to the internal compartment (endoplasmic reticulum, PIN5) or to both the internal compartment and the PM (PIN8) [3-5].

Long PINs show diverse polar localization in the PM of diverse cell types [6-11]. Although de novo synthesized long PINs symmetrically localize to the PM, subsequent cycles of endocytosis and directional recycling (exocytosis) processes determine and maintain specific PIN polarity [12,13]. The endocytosis/recycling process of long PINs is coordinated and regulated by environmental and cellular signals [13,14].

Protein phosphorylation and dephosphorylation have been implicated in auxin transport and PIN trafficking [13]. There is no direct evidence of a role for the TM domain in auxin transport by PINs, but several lines of evidence support the idea that the HL acts in regulation of intracellular trafficking of PIN proteins. The HL of long PINs contains several phosphorylation motifs that are targeted by some kinases. Protein kinase inhibitors suppressed cellular auxin transport [15,16], and interfered with the trafficking of PIN proteins to the PM [16]. Mutation or overexpression of PINOID (PID, a serine/threonine [S/T] protein kinase) changed auxin transport activity and led to phenotypes associated with auxin transport defects [16-18]. It has been proposed that changes in phosphorylation status by the antagonistic activities between PID (and related kinases) and the protein phosphatase PP2A modulate the subcellular polarity of PINs [19,20]. Accordingly, it has been demonstrated that the PIN-HL can be phosphorylated by PID and related kinases *in vitro* and in a protoplast phosphorylation assay, and that the phosphorylation sites of the PIN-HL are required for PINs’ polarity and biological functions [20-24].

Recently, we reported a phosphorylatable motif, named M3, in the PIN3-HL that includes five S/T (four S and one T) residues and is necessary for the polarity and intracellular trafficking of PIN3 [25]. Root hair-specific overexpression of wild-type PIN3 greatly inhibited root hair growth by exporting auxin from the root hair cell, whereas overexpression of a phosphorylation-defective M3 mutant PIN3 failed to inhibit root hair growth. To restore root hair growth, at least three phosphorylation-defective mutations among the five S/T residues were required. The PM localization of PIN3 in root hair cells was disrupted and internalized by the phosphorylation-defective mutations because of the defect in PM targeting. The effect of M3 phosphorylation-defective mutation on the subcellular localization of PIN3 was also observed in its native expression domain, root pericycle cells, resulting in aberrant localization to the outer lateral PM and defects in gravitropism. The M3 motif sequence is conserved among all long PINs. In this study, using the root hair assay system for PIN activity, we investigated whether this motif has been functionally conserved among long PINs.

## Results

To examine the functional conservation of the M3 motif among long PINs, we chose PIN7 (closest to PIN3, Additional file 1: Figure S1A) and two other well-studied long PINs, PIN1 and PIN2. The five phosphorylatable S/T residues in the M3 motif are strictly conserved with very few exceptions among long PINs including PIN1, 2, and 7 ([25], Additional file 1: Figure S1B). Most of the M3 S/T residues from PIN1, 2, and 7 are predicted to be phosphorylatable by NetPhos 2.0 (http://www.cbs.dtu.dk/services/NetPhos/, [26]), as are the PIN3 residues (Additional file 1: Figure S1C). PIN2 shows a unique feature in that its M3 motif lacks one putative phosphorylation serine that is replaced by alanine (A212 in PIN2, Additional file 1: Figure S1C). While the M3 phosphorylation sites are conserved, there is diversity in the sequence between the phosphorylation sites. These features of M3 in long PINs indicate both conservation and probably specific divergence in function.

### The M3 motif is required for the auxin efflux activity of PIN1, PIN2, and PIN7 in the Arabidopsis root hair cell

In a previous study, we divided the M3 S/T sites into two sub-motifs, 3 m1 comprising the first three S residues and 2 m3 comprising the last two T/S residues (Additional file 1: Figure S1B). Mutation of the 3 m1 S residues caused defects in the phosphorylation, trafficking, efflux activity, and biological functions of PIN3, but 2 m3 mutation did not [25]. To assess the function of the M3 motif in the auxin efflux activity of PIN1, PIN2, and PIN7, we generated phosphorylation-defective (S to A) M3 (all five S/T residues) and 3 m1 (first three S residues) mutant PIN:GFP (green fluorescent protein) fusion constructs, which were root-hair specifically expressed under the *EXPANSIN A7* promoter (ProE7, [27,28]) in Arabidopsis plants. As previously reported, this root hair system was adopted to indirectly estimate the PINs’ auxin efflux activity because root hair growth depends on auxin levels in the root hair cell [3,16,29,30].

**Figure 1:**
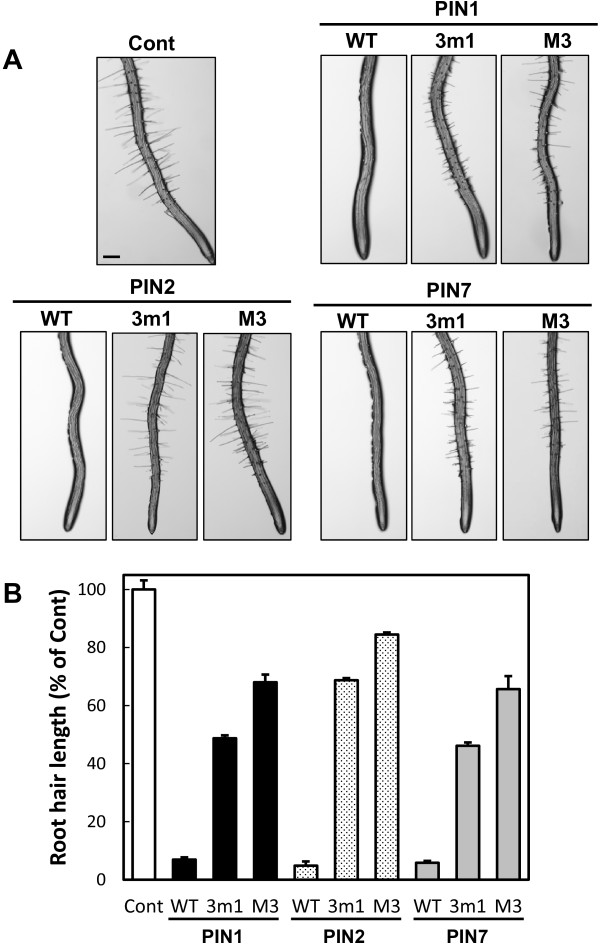
**M3 motif is required for PIN activities in root hair cells. (A)** Representative root images of the control (ProE7:YFP), wild-type PIN expression lines (WT), and phosphorylation-defective PIN expression lines (3 m1 and M3). WT-, 3 m1-, and M3-PINs were expressed under the root hair-specific EXPASIN A7 promoter (ProE7). Bar = 0.1 mm for all. **(B)** Root hair lengths of PIN-expressing transformants. The results were obtained from 22–59 seedlings/414–2332 root hairs from five independent lines. Data represent means ± SE.

Consistent with our previous report [3], root hair-specific overexpression of wild-type PIN1, PIN2, or PIN7 greatly inhibited the root hair growth of 4-d-old seedlings by depleting auxin levels in the root hair cell (Figure 1). On the other hand, this PIN-mediated root hair inhibition was significantly suppressed by 3 m1 or M3 mutation, though M3 mutation consistently showed a greater restoration of root hair growth than did 3 m1 mutation (Figure 1), similarly to previous results with PIN3 [25]. Among the three PINs, the 3 m1 and M3 mutants of PIN2 showed the greatest restoration of root hair growth (Figure 1B). These results were reproducible with 15 independent transgenic lines for each PIN (Additional file 1: Figure S2).

In the case of PIN3, 3 m1 and M3 mutations suppressed the auxin efflux activity, and thus root hair inhibition, of PIN3 by interrupting its trafficking to the PM [25]. To test whether this was the same for PIN1, PIN2, and PIN7, we examined the subcellular localization of the mutant forms of these PINs.

### The M3 motif is required for PM targeting of PIN1 and PIN7 in the root hair cell

**Figure 2:**
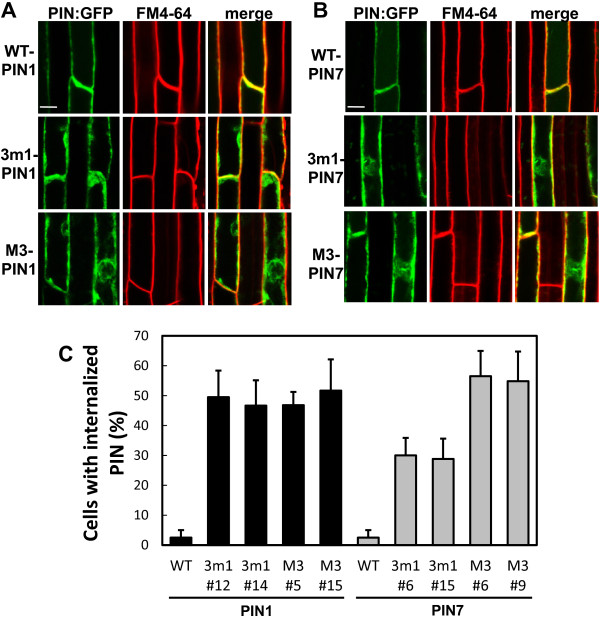
**Phosphorylation-defective mutations in the M3 motif of PIN1 and PIN7 partly disrupted their PM localization in root hair cells. (A)** and **(B)** Confocal images showing the subcellular localization of ProE7:PIN:GFP (WT-PIN), ProE7:3 m1-PIN:GFP (3 m1-PIN1), and ProE7:M3-PIN:GFP (M3-PIN) in root hair cells. Transgenic seedlings were observed with FM4-64 staining (2 μM, within 3 min). Representative images are shown. Bar = 10 μm in all cases. **(C)** Statistical analysis for the internalization of mutant PIN1 and PIN7 proteins. The ratios of root hair cells containing internally-localized PIN proteins were calculated with 29–36 total root hair cells from 10 seedlings for each transgenic line. Data represent means ± SE.

To determine whether the mutant PINs were PM or internally localized, GFP-tagged PINs were first co-visualized with the endocytic tracer FM4-64 in the root hair cell. Wild-type PIN1 and PIN7 overlapped with the FM4-64 signal at the PM, whereas the 3 m1- and M3-mutant forms of PIN1 and PIN7 showed considerable internal localization, though they were partly localized in the PM as well (Figure 2). This internal localization pattern of mutated PIN1 and PIN7 was consistently observed in 10 independent transgenic lines (Additional file 1: Figures S3 and S4). Observation of two representative transgenic lines of 3 m1- and M3-PINs showed that PIN1 internalization occurred in ~50% of the root hair cells, whereas, in the case of PIN7, internalization occurred in ~30% of 3 m1 and ~55% of M3 mutant transgenic lines under similar conditions (Figure 2C). These data support the idea that the probable phosphorylation-defective mutations in the 3 m1 or M3 motifs significantly impaired the capability of PIN1 and PIN7 to target to the PM and thus resulted in suppression of PIN-mediated root hair inhibition.

**Figure 3:**
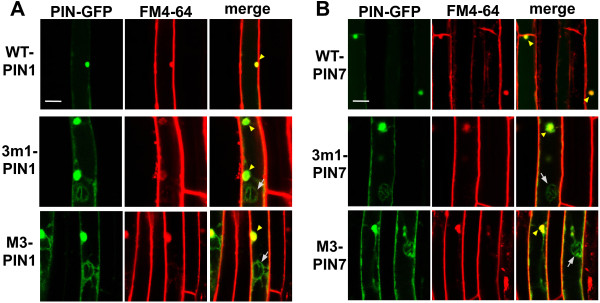
**M3 phosphorylation-defective PIN proteins partially co-localized with BFA compartments in root hair cells. (A)** and **(B)** Confocal images showing the subcellular localization of ProE7:PIN:GFP (WT-PIN), ProE7:3 m1-PIN:GFP (3 m1-PIN1), and ProE7:M3-PIN:GFP (M3-PIN) in root hair cells after BFA treatment. Transgenic seedlings were pretreated with cycloheximide (50 μM, 30 min), followed by BFA (25 μM, 1 h), and stained with FM4-64 (2 μM). Phosphorylation-defective PIN proteins either co-localized with the BFA compartments (arrow heads) or did not (arrows). Representative images are shown. Bar = 10 μm in all cases.

The exocytosis inhibitor brefeldin A (BFA) causes internal accumulation of PIN proteins into so-called ‘BFA compartments’, which are indicative of PM-localization of PINs [31,32]. To clarify the subcellular localization of mutant PIN1 and PIN7 proteins, the transgenic seedlings were co-treated with the protein synthesis inhibitor cycloheximide (CHX) and BFA. Signals of wild-type PIN1 and PIN7 proteins completely overlapped with those of FM4-64 in the BFA compartments, whereas 3 m1 or M3 mutant PIN1 and PIN7 proteins only partly co-localized with the FM4-64-stained BFA compartments (Figure 3 and Additional file 1: Figure S5). The lack of internal mutant PIN signal and BFA compartment overlap further supports the idea that the ability of PIN1 and PIN7 to target to the PM was partially disrupted by the 3 m1 and M3 phosphorylation-defective mutations.

### M3 mutations lead to rapid degradation of PIN2 through the vacuolar lytic pathway in the root hair cell

**Figure 4:**
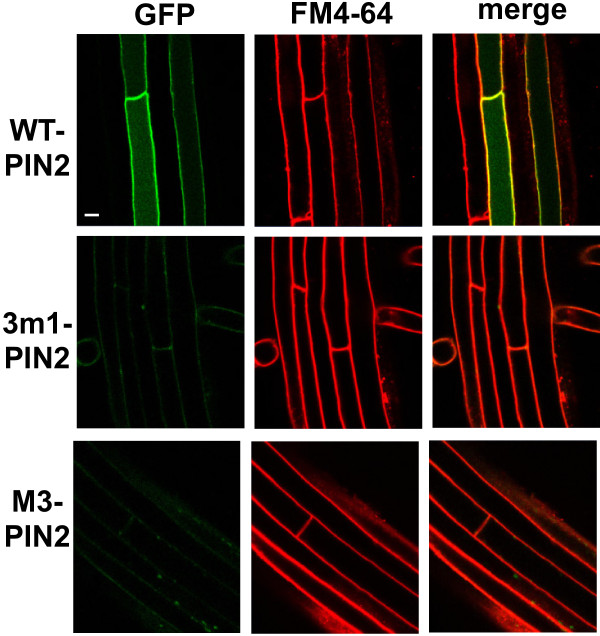
**M3 mutations greatly decreased cellular PIN2 levels in root hair cells.** Confocal images showing the subcellular localization of ProE7:PIN2 (WT-PIN2), ProE7:3 m1-PIN2, and proE7:M3-PIN2 in root hair cells. Transgenic seedlings were stained with FM4-64 (2 μM). Representative images are shown. Bar = 10 μm in all cases.

Unlike phosphorylation-defective PIN1 and PIN7, the mutant PIN2 proteins showed completely different trafficking behavior. While wild-type PIN2 was clearly localized to the PM, phosphorylation-defective 3 m1- or M3-PIN2 proteins were barely detectable in root hair cells; this was consistent in all five independent transgenic lines observed for each mutant construct (Figure 4 and Additional file 1: Figure S6A). This result was in accordance with the results from the root-hair-inhibition assay, where 3 m1 or M3 mutant PIN2s showed much weaker inhibitory effects (~30% and ~15% inhibitory effects for 3 m1 and M3, respectively) on root hair growth than did mutant PIN1 or PIN7 (Figure 1B).

The very low expression of these mutant PIN2 proteins in the cell could be due to either translational failure or degradation of the mutant proteins because the mutant transgenes seemed to be normally transcribed, as shown in the RT-PCR analysis (Additional file 1: Figure S6B). Because there were some residual inhibitory effects on root hair growth as mentioned above, it is conceivable that a certain portion of mutant PIN2 proteins might stay transiently in the PM, resulting in weak auxin-exporting activity, and then undergo endocytosis and degradation. To test this idea and to restore the mutant PIN2 protein in the cell, we applied auxin (1-naphthaleneacetic acid, NAA) to inhibit clathrin-dependent PIN endocytosis from the PM [33], MG132 to inhibit ubiquitylation/proteasome-mediated PIN internalization and degradation [34,35], and wortmannin to inhibit the vacuolar lytic pathway [36].

**Figure 5:**
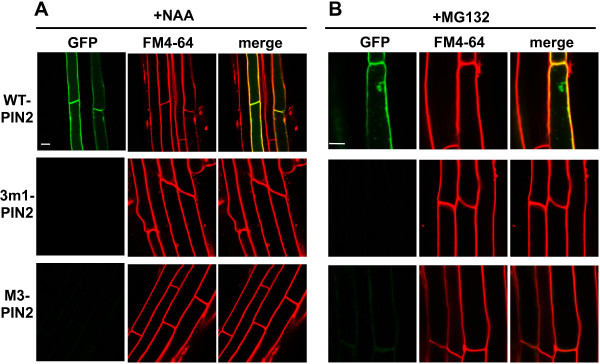
**M3 phosphorylation-defective PIN2 proteins could not be restored by auxin or MG132 in root hair cells. (A)** M3 phosphorylation-defective PIN2 proteins were not restored by exogenous auxin in root hair cells. GFP signals of ProE7:PIN2:GFP, ProE7:3 m1-PIN2:GFP, and ProE7:M3-PIN2:GFP were visualized. Transgenic seedlings were treated with 5 μM NAA for 2 h and stained with FM4-64 (2 μM). Representative images are shown. Bar = 10 μm in all cases. **(B)** M3 phosphorylation-defective PIN2 proteins were not restored by MG132 treatment in root hair cells. Transgenic seedlings were treated with 25 μM MG132 for 2.5 h and stained with FM4-64. Representative images are shown. Bar = 10 μm in all cases.

**Figure 6:**
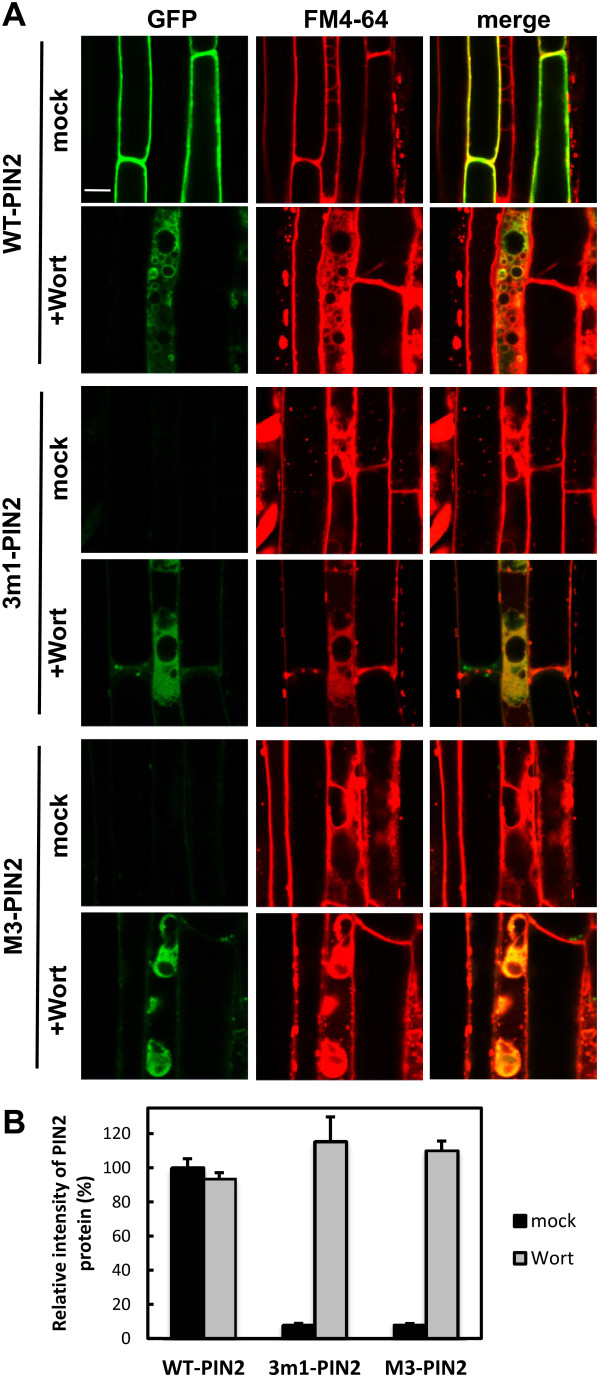
**Wortmannin restored M3 phosphorylation-defective PIN2 proteins in root hair cells. (A)** Confocal images showing the subcellular localization of ProE7:PIN2, ProE7:3 m1-PIN2, and ProE7:M3-PIN2 in root hair cells. Transgenic seedlings were treated with 20 μM wortmannin (Wort) for 12 h and stained with FM4-64 (2 μM, for 2 h). Representative images are shown. Bar = 10 μm in all cases. **(B)** Relative intensities of PIN2:GFP signals in root hair cells from 7–9 seedlings. Values are relative to the WT-PIN2 mock treatment. Data represent means ± SE.

NAA (5 μM, for 2 h) did not have much effect on the PM-localization of wild-type PIN2 or restored 3 m1- or M3-PIN2 proteins in the root hair cell (Figure 5A and Additional file 1: Figure S7). MG132 (25 μM, for 2.5 h) caused partial internal accumulation of wild-type PIN2 proteins, but failed to recover the mutant PIN2 signals in the root hair cell (Figure 5B and Additional file 1: Figure S8). Wortmannin, an inhibitor of phosphatidylinositol-3-kinase, inhibits the trafficking of PIN proteins as well as FM4-64 to the vacuole [13]. Wortmannin treatment (12 h pretreatment and 2 h with FM4-64) restored the GFP signals of 3 m1 and M3 mutant PIN2:GFP proteins in the root hair cell (Figure 6 and Additional file 1: Figure S9). When five independent lines for each of two mutant PIN2 constructs were observed, the intensities of wortmannin-restored mutant PIN2 signals were similar to those of wild-type PIN2 (Figure 6 and Additional file 1: Figure S9), implying that the translational efficiency of the wild-type and mutant PIN2s was similar, but that the 3 m1 or M3 phosphor-defective PIN2 proteins tended to enter the lytic pathway more than the wild-type protein.

## Discussion

In this study, we investigated whether the M3 motif, a phosphorylation motif originally found in the PIN3-HL that regulates PIN3’s polarity and intracellular trafficking [25], has been functionally conserved in other long-looped PINs using the root hair assay system to estimate PIN activity and by observing intracellular PIN trafficking. In the four Arabidopsis long-looped PINs (PIN1, 2, 3, and 7) investigated in this and previous studies, the M3 motif appears to be generally necessary for proper intracellular trafficking of these long PINs.

Among Arabidopsis PIN members, PIN7 is most closely related to PIN3 (Additional file 1: Figure S1A), and their HL amino acid sequences share 74% identity. Previous studies have reported that both PIN3 and PIN7 proteins are expressed in the same tissues of the Arabidopsis root with the same polarity, such as basally polar in stele cells [6,8] and apolar in columella cells [8,9]. These observations suggest that the intracellular behaviors of both PIN3 and PIN7 could be regulated by similar intracellular trafficking machinery. This led us to hypothesize that the function of the M3 motif in PIN7-HL would be comparable to that in PIN3-HL for the regulation of its trafficking. Our previous study showed that phosphorylation-defective M3-PIN3 was completely internally localized (to vacuoles) in the root hair cell [25]. However, the M3-PIN7 proteins in this study were partly internally localized, and a considerable amount of mutant PIN7 remained in the PM (Figure 2 and Additional file 1: Figures S3 and S4). In accordance with its partial PM localization, the mutant PIN7 proteins showed a different response to BFA from that of M3-PIN3 in the root hair cell; namely, no overlap of mutant PIN3 [25] but partial overlap of mutant PIN7 proteins with the BFA compartments (Figure 3 and Additional file 1: Figure S5).

The M3 motifs of the three long PIN subgroups (‘PIN3, 7, and 4 cluster’, ‘PIN1’, and ‘PIN2’) show distinctive structures (Additional file 1: Figure S1, [25]). Compared with PIN1 and PIN2, the PIN3, 7, and 4 cluster members have a gap of 6–7 amino acid residues between S212 and S215. Even among PIN3, 7, and 4 cluster members, although the residues between S209 and S215 are highly conserved, those between S215 and T222 show considerable sequence divergence (Additional file 1: Figure S1, [25]). Furthermore, although the expression patterns and subcellular polarities of PIN3 and PIN7 are mostly alike, some minor differences in their subcellular polarities have been found. For example, in the root pericycle cell, PIN3 is localized to the inner lateral and basal sides [8,25], whereas PIN7 shows no polarity in the same cell type [37]. The partially different trafficking behaviors between M3-PIN3 and M3-PIN7 in the root hair cell could be due to the minor divergence in the M3 motif.

PIN2 undergoes active degradation under normal growth conditions or in response to environmental stimuli such as gravi-stimulation or dark conditions, and its lytic vacuolar targeting and degradation are associated with ubiquitylation/proteasome activity [12,34,35,38-40]. These findings imply that diverse molecular cues in PIN2 are involved in the regulation of its degradation responses. These molecular cues might be located in the PIN2-HL, and this study suggests that the M3 motif could be one of them. Several lines of evidence from our results support the idea that the M3 motif of PIN2 is involved in PIN2 degradation, in particular, by modulating the rate of vacuolar lytic trafficking at least in the root hair cell. First, 3 m1 or M3 mutation of PIN2 decreased cellular PIN2 proteins to an almost undetectable level (Figure 4 and Additional file 1: Figure S6A). Second, the mutation effect on the PIN2 protein level seems to be due to degradation but not transcription or translation of PIN2, because the transcription of mutant PIN2 genes was normal (Additional file 1: Figure S6B) and the wortmannin-restored protein levels of mutant PIN2 were similar to those of wild-type PIN2 (Figure 6 and Additional file 1: Figure S9). Third, 3 m1 or M3 mutant PIN2 proteins were restored in the root hair cell by wortmannin (an inhibitor of the vacuolar lytic pathway), but not by auxin (an inhibitor of clathrin-dependent endocytosis). Because similar protein levels were observed between wild-type and mutant PIN2 upon wortmannin treatment but mutant PIN2 proteins were undetectable without wortmannin (Figure 6 and Additional file 1: Figure S9), it is likely that mutations in the M3 phosphorylation sites facilitated the trafficking of mutant PIN2 proteins to the vacuolar lytic pathway.

On the other hand, the result with MG132 is intriguing in that it was not able to inhibit the lytic vacuolar pathway of M3- (and 3 m1) PIN2. This proteasome inhibitor was found to inhibit the internalization or vacuolar targeting of PIN2 from the PM [34,35], and ubiquitylation of PIN2 is implicated in this process [38]. So far, it remains uncertain how proteasome-mediated degradation contributes to PIN2 trafficking to the lytic vacuole and whether PIN2 ubiquitylation is directly linked to proteasome-mediated degradation. Proteasomes are localized in the nucleus and cytoplasm [41], and vacuole-mediated protein degradation is distinct from proteasome-mediated protein degradation [42]. In this context, we can propose two possibilities. First, ubiquitylation of PIN2 might act as a signal for vacuolar targeting but not for proteasome-mediated cytoplasmic degradation. In this case, there could be some cytoplasmic protein factors that are proteasome-sensitive and engaged in the vacuolar trafficking of ubiquitylated PIN2. Second, cytoplasmic proteasomes and vacuolar proteases might both work together for PIN2 degradation in a similar way to the degradation of the a-factor transporter (Ste6p) in yeast [42]. The first possibility is more feasible because the inhibitory effect of MG132 is more obvious in PIN2 endocytosis and vacuolar trafficking [34,35]. The inability of MG132 to block the vacuolar trafficking of M3- (3 m1)-PIN2 in this study prompts us to speculate that phosphorylation of the M3 (3 m1) motif could be necessary for normal vacuolar trafficking of PIN2, which is regulated coordinately with other factors such as ubiquitylation of the PIN2-HL and proteasome-sensitive trafficking components. Impairment of M3 (3 m1) phosphorylation might disrupt the regulated vacuolar trafficking and redirect PIN2 trafficking directly to the vacuole.

Overall, our results suggest that the M3 motif in PIN2 may have specialized to modulate protein degradation by lytic vacuolar trafficking. The aforementioned fact that PIN2 protein degradation is frequently observed under various conditions may reflect that PIN2 is more susceptible to the protein trafficking machinery for degradation. The different behavior of M3- (and 3 m1) PIN2 from that of other M3-PINs leads us to hypothesize that the specialized function of the PIN2 M3 motif could be due to its specific structure. The M3 motif of PIN2 not only has additional amino acid residues between S212 and S215, but also includes additional serine residues in the M3 motif other than the five S/T residues conserved in the long PIN M3 motif (Additional file 1: Figure S1B, [25]). Furthermore, S212 of the Arabidopsis PIN2 M3 motif is replaced by alanine. It is conceivable that these unique features of PIN2 M3 might drive PIN2 trafficking more frequently to the vacuolar lytic pathway.

Including the M3 motif, at least three phosphorylation motifs have been characterized from long PIN HL domains [43]. The serine residue in the ‘TPRXS’ (where X is any amino acid residue) motif of PIN1- and PIN2-HLs has been shown to be phosphorylated and function in PIN polarity [22,23]. Long PIN HLs have three repeats of TPRXS, mutations of which additively affect developmental phenotypes [23]. Another motif, ‘Ser337/Thr340’, is required for the proper polarity and biological function of PIN1 [44].

The M3 motif consists of 3 m1 and 2 m3 regions, with the 2 m3 region corresponding to the first TPRXS motif (Additional file 1: Figure S1, [25]). Our previous study showed that mutation of 3 m1 (with three serine residues) alone in PIN3 was sufficient to cause phenotypic effects such as alteration of its PM targeting, polarity, and biological function [25]. Conversely, 2 m3 mutation alone did not have any detectable effect on such phenotypes [25]. Furthermore, 3 m1 mutation affected PID- or WAG1-mediated phosphorylation of PIN3-HL whereas 2 m3 mutation did not [25]. Our previous results with 2 m3 mutant PIN3 were similar to results with PIN1 mutated at the first TPRXS [22], but different from results with PIN2, where the mutation of S in the first TPRXS significantly decreased PID- or WAG-mediated phosphorylation of PIN2 and affected developmental phenotypes [23]. These results imply that the same TPRXS motif from different PINs may have different affinity or specificity to different kinases [43]. Although mutations of 3 m1 and 2 m3 together had a much greater effect than 3 m1 mutation alone, the 3 m1 mutation alone had a considerable effect on the trafficking behavior and activity of long PINs (Figures 1, 2, and 4, [25]). These data suggest that the 3 m1 motif provides long PINs with a partly independent phosphorylation code for the modulation of their subcellular trafficking. However, it should be noted that our current results were based on the root hair model system. Studying individual PINs in the cells in which they are most expressed should help identify the role of the 3 m1 motif in native PIN trafficking and plant development. Previous studies and this study collectively suggest multiple phosphorylation sites in long PIN-HLs, divergent functions of conserved phosphorylation sites among different PINs, and differential targeting by different AGC kinases. In addition, considering the different cell type-specific expression patterns among different PINs and kinases, various combinations of the phosphorylation code on the PIN-HL would modulate the cell type- and molecule-specific trafficking behavior of long PINs.

## Conclusions

Our results demonstrate that the M3 motif of long PINs has been functionally conserved for intracellular PIN trafficking. Moreover, divergence in the M3 structure among long PINs has likely lead to divergence in its specific role in PIN trafficking, so that the mutation of M3 caused complete (for PIN3) or partial (for PIN1 and PIN7) failure of PM targeting and facilitation of vacuolar lytic trafficking (for PIN2). The dynamicity of subcellular PIN polarity is important to modulate local auxin gradient formation and ultimately diverse developmental processes, which are subject to perpetual changes in environmental and developmental stimuli. Phosphorylation of the PIN-HL domain provides PINs the ability to change their subcellular trafficking behavior. Furthermore, multiple phosphorylation sites in the PIN-HL domain and their divergence in specific structure may generate diverse phosphorylation codes to drive dynamic PIN trafficking depending on internal and external stimuli.

## Methods

### Plant materials and growth conditions

Arabidopsis (*Arabidopsis thaliana*) Columbia ecotype (Col-0) was used as the wild-type plant in this study. All seeds were grown on agarose plates containing half-strength Murashige and Skoog (MS) nutrient mix (Sigma-Aldrich), 1% sucrose, 0.5 g/L MES (pH 5.7), and 0.8% agarose. All seeds were cold treated (4°C) for 3 days and germinated at 22°C under a 16-h-light/8-h-dark photoperiod. *Arabidopsis* transformation was accomplished by *Agrobacterium tumefaciens* (strain C58C1)-mediated infiltration [45]. Transformed plants were selected on hygromycin-containing plates (10 mg/mL). For root hair length estimation and microscopic analyses, T2 plants were observed.

### Transgene constructs

The binary vector pCAMBIA1300-NOS with modified cloning sites [46] was used for transgene construction. The *Arabidopsis* EXPANSIN A7 promoter (ProE7, [27,28]) was used for root hair-specific expression of PIN genes. The ProE7:YFP, ProE7:PIN1:GFP, ProE7:PIN2:GFP, and ProE7:PIN7:GFP constructs were described previously [3,16]. Site-directed mutagenesis of the putative phosphorylation sites of PIN1-HL, PIN2-HL, and PIN7-HL to generate ProE7:3 m1-PIN:GFP and ProE7:M3-PIN:GFP was performed by the PCR method using ProE7:PIN:GFP constructs, mentioned above, as templates and the primers listed in Additional file 1: Table S1. Those phosphorylation-mutated PIN:GFP fragments were cloned into the designated enzyme sites (Additional file 1: Table S1) of the binary vector containing the ProE7 fragment.

### Measurement of root hair length

The root hair lengths of 4-day-old seedlings were measured as described previously [47]. Digital photographs of roots were taken under a microscope (DFC425C, Leica) at 40× magnification. The root hair lengths of 30 to 45 hairs from both sides of the root were measured.

### Microscopic observation and quantification of PIN-GFP signals

GFP (green) and FM4-64 (red) fluorescence were observed using a LSM700 confocal laser scanning microscope (Carl Zeiss). Green and red fluorescence were detected by 488/490–555 nm and 555/640 excitation/emission filter sets, respectively. Localization of PIN:GFP fusion proteins was observed in 4-day-old seedlings. For observation of the subcellular localization of PIN:GFP after BFA, MG132, auxin, or wortmannin treatment, seedlings were incubated in half-strength liquid MS medium for the indicated time periods. The control (mock) liquid medium included the same amount of the solvent dimethylsulfoxide (0.04%). FM4-64 (2 μM), BFA (25 μM), CHX (50 μM), MG132 (25 μM), NAA (5 μM), or wortmannin (20 μM) was applied to the seedlings before observation as described in the results. The relative intensity of PIN2:GFP signals with or without wortmannin was estimated from the root hair cells of 7–9 seedlings using the Adobe Photoshop histogram menu as described previously [27,48].

### Accession numbers

The Arabidopsis Information Resource (TAIR) accession number for the genes mentioned in this article are At1G12560 (EXPANSIN A7), At1G73590 (PIN1), At5G57090 (PIN2), At1G70940 (PIN3), At2G01420 (PIN4), At5G16530 (PIN5), At1G77110 (PIN6), At1G23080 (PIN7), At5G15100 (PIN8).

## Abbreviations

BFA: Brefeldin A; CHX: Cycloheximide; HL: Hydrophilic loop; NAA: 1-naphthaleneacetic acid; PIN: PIN-FORMED; PM: Plasma membrane; TM: Transmembrane.

## Competing interests

The authors declare that they have no competing interests.

## Authors’ contributions

DS performed most experiments. DS and HTC designed experiments, analyzed the data, and wrote the manuscript. AG and MP participated in the confocal work. All authors read and approved the final manuscript.

## Supplementary Material

Additional file 1: Figure S1 Conservation of the M3 motif in long PINs. **Figure S2.** Root hair length of independent transgenic lines. **Figure S3.** Confocal images of M3 phosphorylation-defective PIN1 lines. **Figure S4.** Confocal images of M3 phosphorylation-defective PIN7 lines. **Figure S5.** Confocal images of root hair cells from BFA-treated M3 phosphorylation-defective PIN7 lines. **Figure S6.** Confocal images and expression analysis of M3 phosphorylation-defective PIN2 lines. **Figure S7.** Confocal images of auxin-treated M3 phosphorylation-defective PIN2 lines. **Figure S8.** Confocal images of MG132-treated M3 phosphorylation-defective PIN2 lines. **Figure S9.** Confocal images of wortmannin-treated M3 phosphorylation-defective PIN2 lines. **Table S1.** Primer list. Click here for file
