# Supplementary material for: The M3 phosphorylation motif has been functionally conserved for intracellular trafficking of long-looped PIN-FORMEDs in the Arabidopsis root hair cell
Source: BMC Plant Biol. 2013 Nov 26;13:189. doi: 10.1186/1471-2229-13-189 (PMC4222813; doi:10.1186/1471-2229-13-189)
Supplement: Additional file 1: Figure S1 — Conservation of the M3 motif in long PINs. Figure S2. Root hair length of independent transgenic lines. Figure S3. Confocal images of M3 phosphorylation-defective PIN1 lines. Figure S4. Confocal images of M3 phosphorylation-defective PIN7 lines. Figure S5. Confocal images of root hair cells from BFA-treated M3 phosphorylation-defective PIN7 lines. Figure S6. Confocal images and expression analysis of M3 phosphorylation-defective PIN2 lines. Figure S7. Confocal images of auxin-treated M3 phosphorylation-defective PIN2 lines. Figure S8. Confocal images of MG132-treated M3 phosphorylation-defective PIN2 lines. Figure S9. Confocal images of wortmannin-treated M3 phosphorylation-defective PIN2 lines. Table S1. Primer list. [file 1471-2229-13-189-S1.pdf]

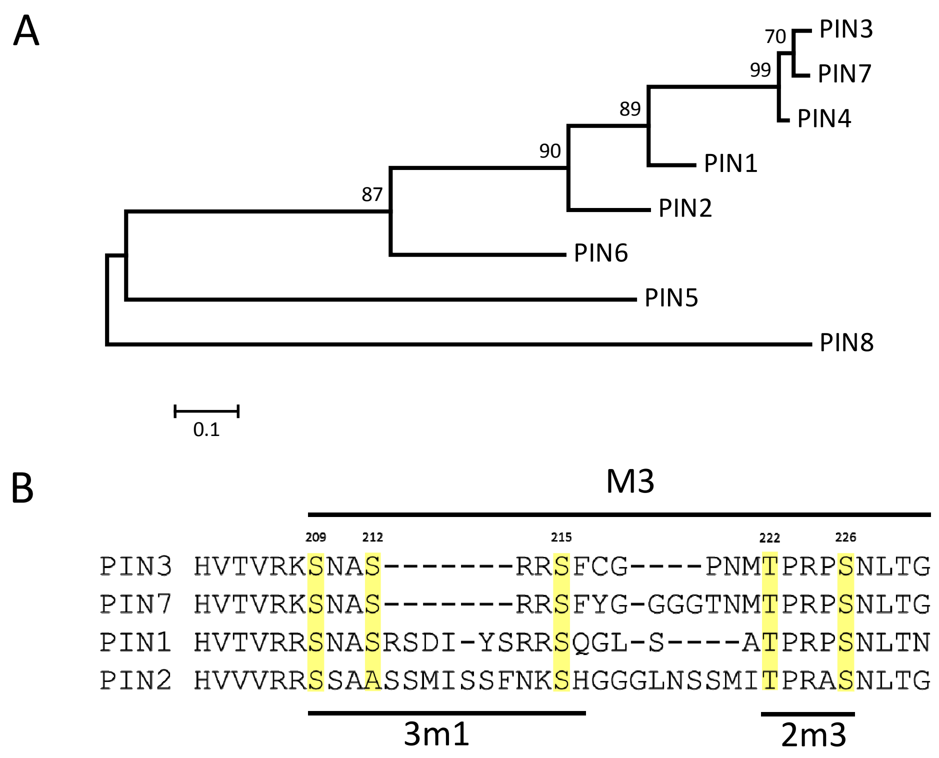

C

	Site1	Score	Site2	Score	Site3	Score	Site4	Score	Site5	Score
PIN3	S209	0.793	S212	0.991	S215	0.716	T222	0.984	S226	0.673
PIN7	S209	0.793	S212	0.989	S215	0.977	T225	0.975	S229	0.673
PIN1	S209	0.333	S212	0.996	S221	0.994	T227	0.861	S231	0.373
PIN2	S209	0.910	A212	-	S222	0.954	T233	0.928	S237	0.827

Figure S1. Conservation of the M3 motif in long PINs. (**A**) Phylogeny of Arabidopsis PIN proteins. The tree was constructed with the HL sequences using the Nighbor-Joining method. The optimal tree with the sum of branch length = 3.53854304 is shown. The percentage of replicate trees in which the associated taxa clustered together in the bootstrap test (1000 replicates) are shown next to the branches. The tree is drawn to scale, with branch lengths in the same units as those of the evolutionary distances used to infer the phylogenetic tree. The evolutionary distances were computed using the Poisson correction method and are in the units of the number of amino acid substitutions per site. All positions containing gaps and missing data were eliminated. (**B**) Alignment of the M3 motifs from PIN1, 2, 3 and 7. Positions were numbered according to PIN3 amino acid sequence. All S/T residues are conserved in the motif except PIN2 A212. (**C**) Phosphorylation index scored by NetPhos 2.0 server (http://www.cbs.dtu.dk/services/NetPhos/).

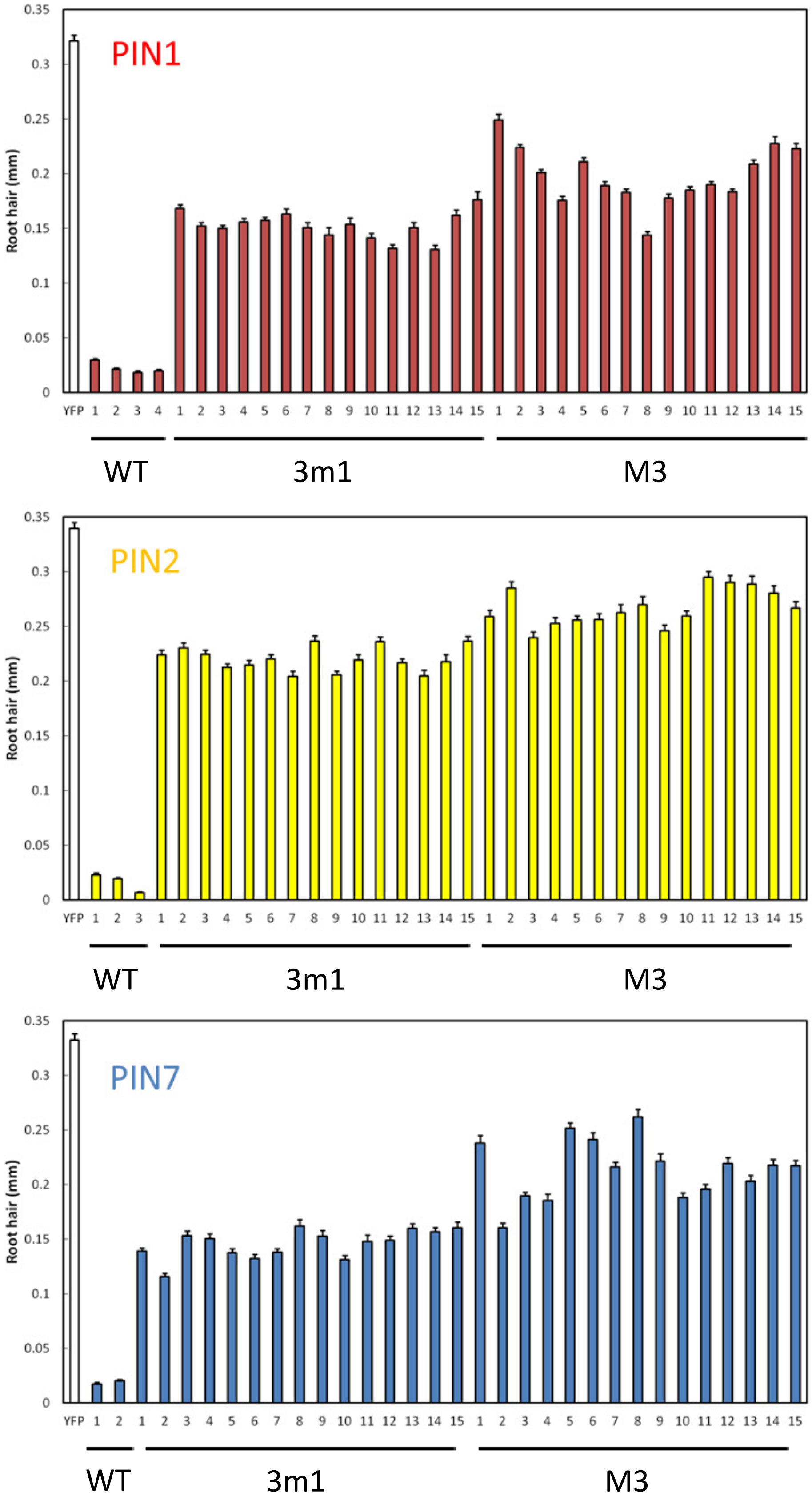

Figure S2. Root hair length of independent transgenic lines. Root hair length was estimated with control (YFP, ProE7:YFP), wild-type PIN expression lines (WT), and phosphorylation-defective PIN expression lines (3m1 and M3). WT-, 3m1-, and M3-PINs were expressed under ProE7. The results were obtained from 7-12 seedlings, 132-485 root hairs for PIN1, 5-16 seedlings, 150-563 root hairs for PIN2, and 5-11 seedlings, 141-453 root hairs for PIN7. Data represent means±SE.

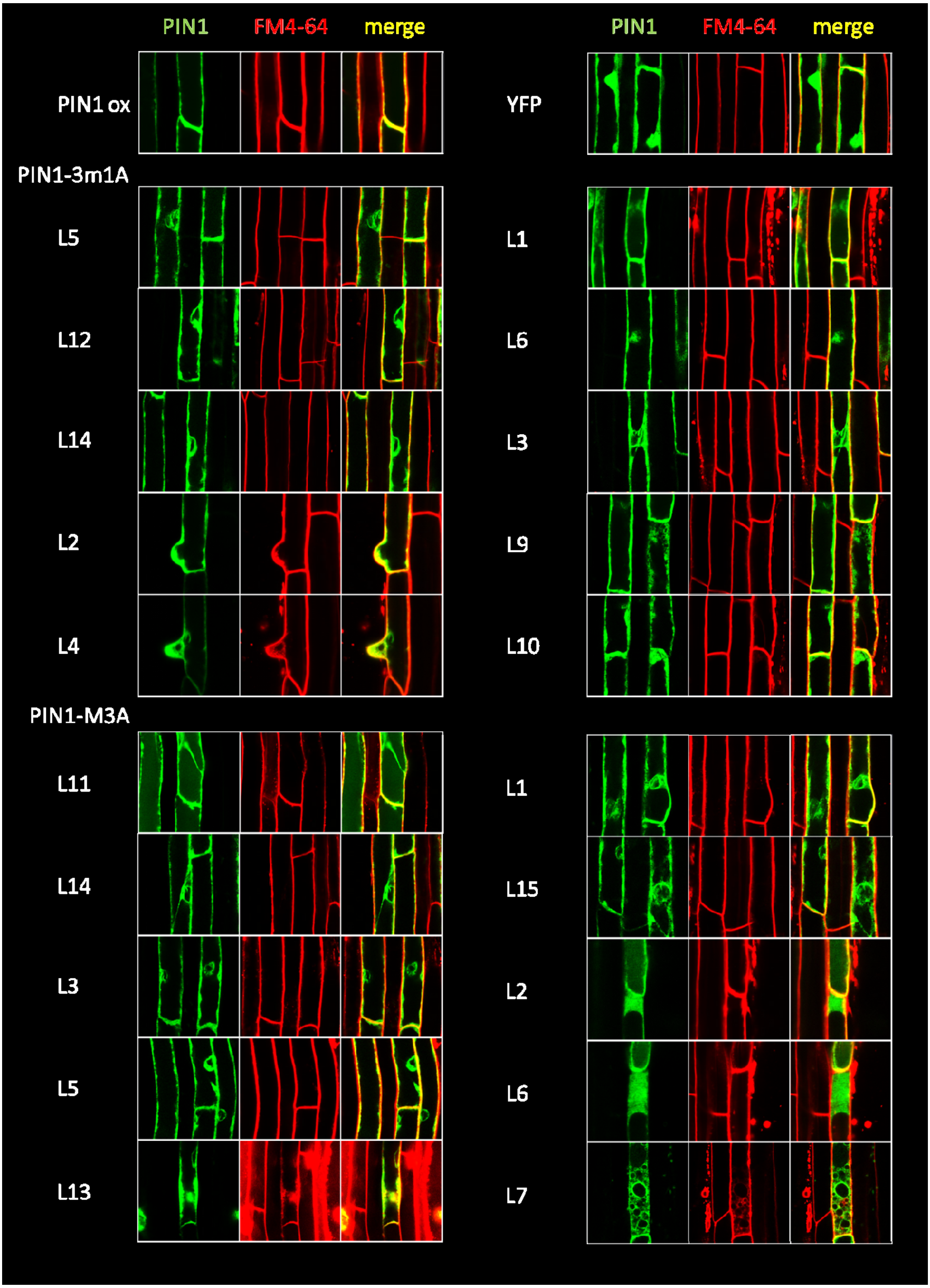

Figure S3. Confocal images of M3 phosphorylation-defective PIN1 lines. Confocal images showing the subcellular localization of ProE7:PIN1, ProE7:3m1-PIN1 (PIN1-3m1A), and ProE7:M3-PIN1 (PIN1-M3A) in root hair cells. Four-day-old seedlings were used and stained with FM4-64 (2 µM). Ten independent lines were observed for each mutant.

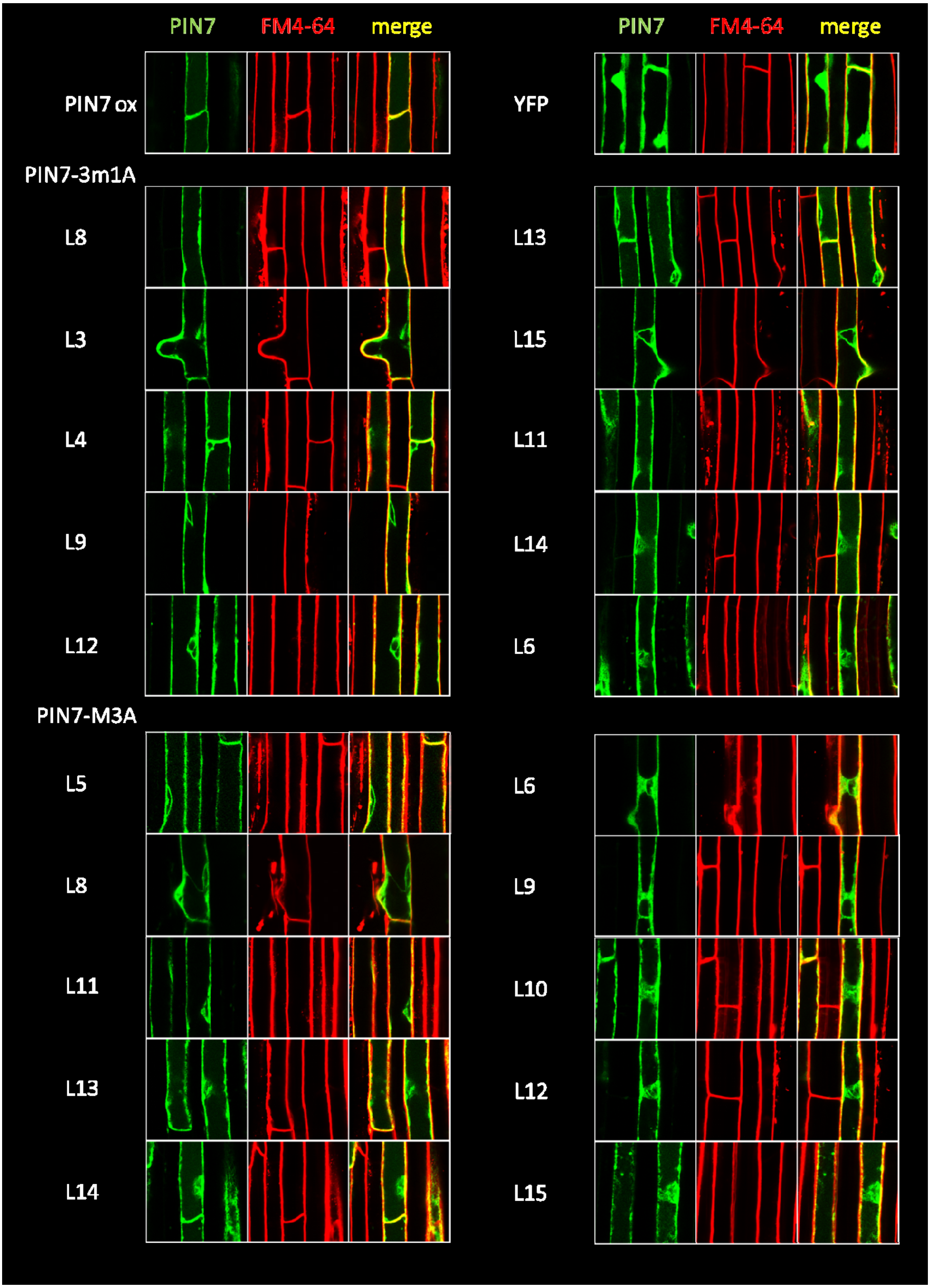

Figure S4. Confocal images of M3 phosphorylation-defective PIN7 lines. Confocal images showing the subcellular localization of ProE7:PIN7, ProE7:3m1-PIN7 (PIN7-3m1A), and ProE7:M3-PIN7 (PIN7-M3A) in root hair cells. Four-day-old seedlings were used and stained with FM4-64 (2 µM). Ten independent lines were observed for each mutant.

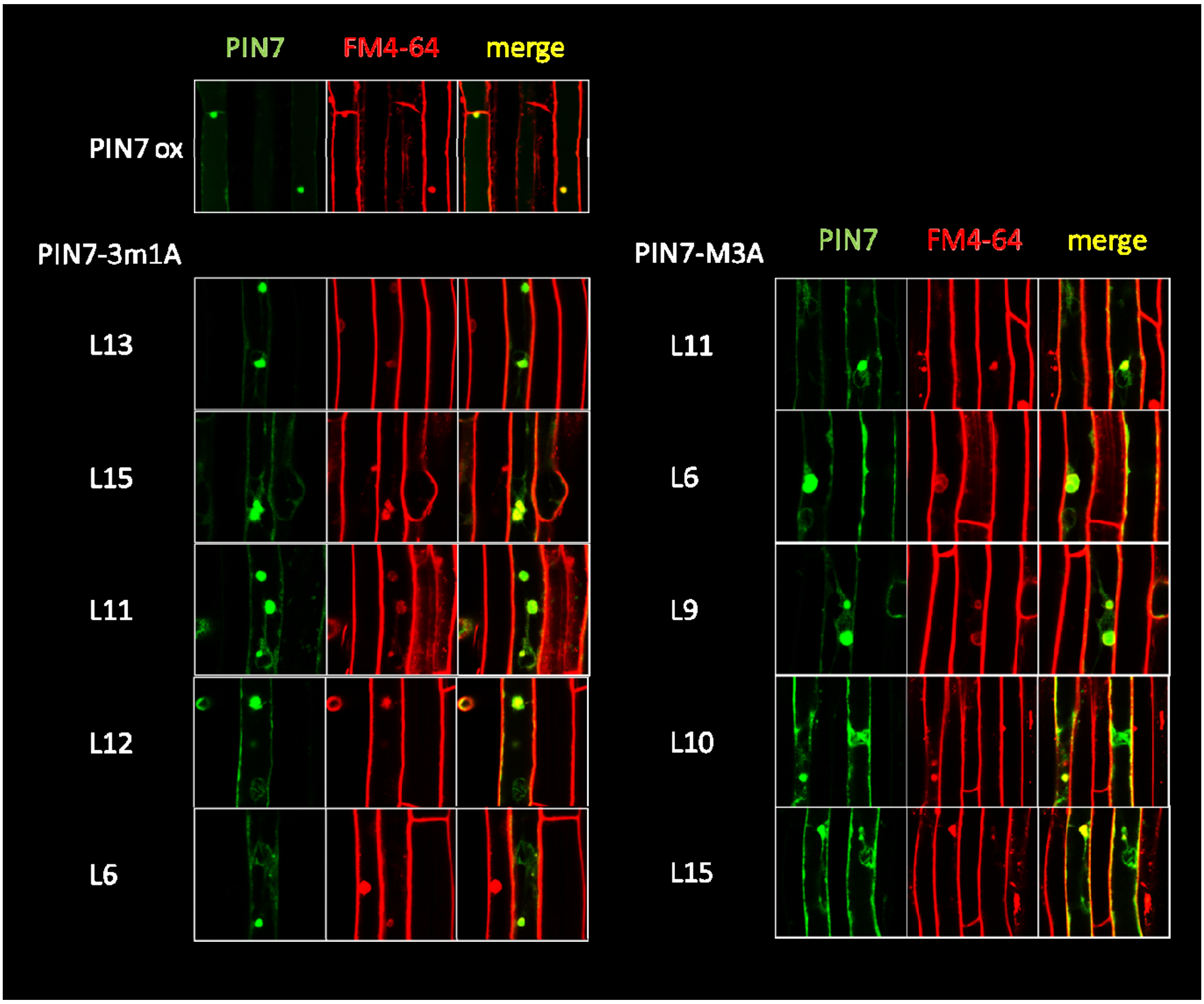

Figure S5. Confocal images of the root hair cells from BFA-treated M3 phosphorylation-defective PIN7 lines. Confocal images showing the subcellular localization of ProE7:PIN7, ProE7:3m1-PIN7 (PIN7-3m1A), and ProE7:M3-PIN7 (PIN7-M3A) in root hair cells after BFA treatment. Transgenic seedlings were pretreated with 50 µM cycloheximide for 30 min, followed by treatment with or without 25 µM BFA for 1 hour, and stained with FM4-64 (2 µM). Five independent lines were observed for each mutant.

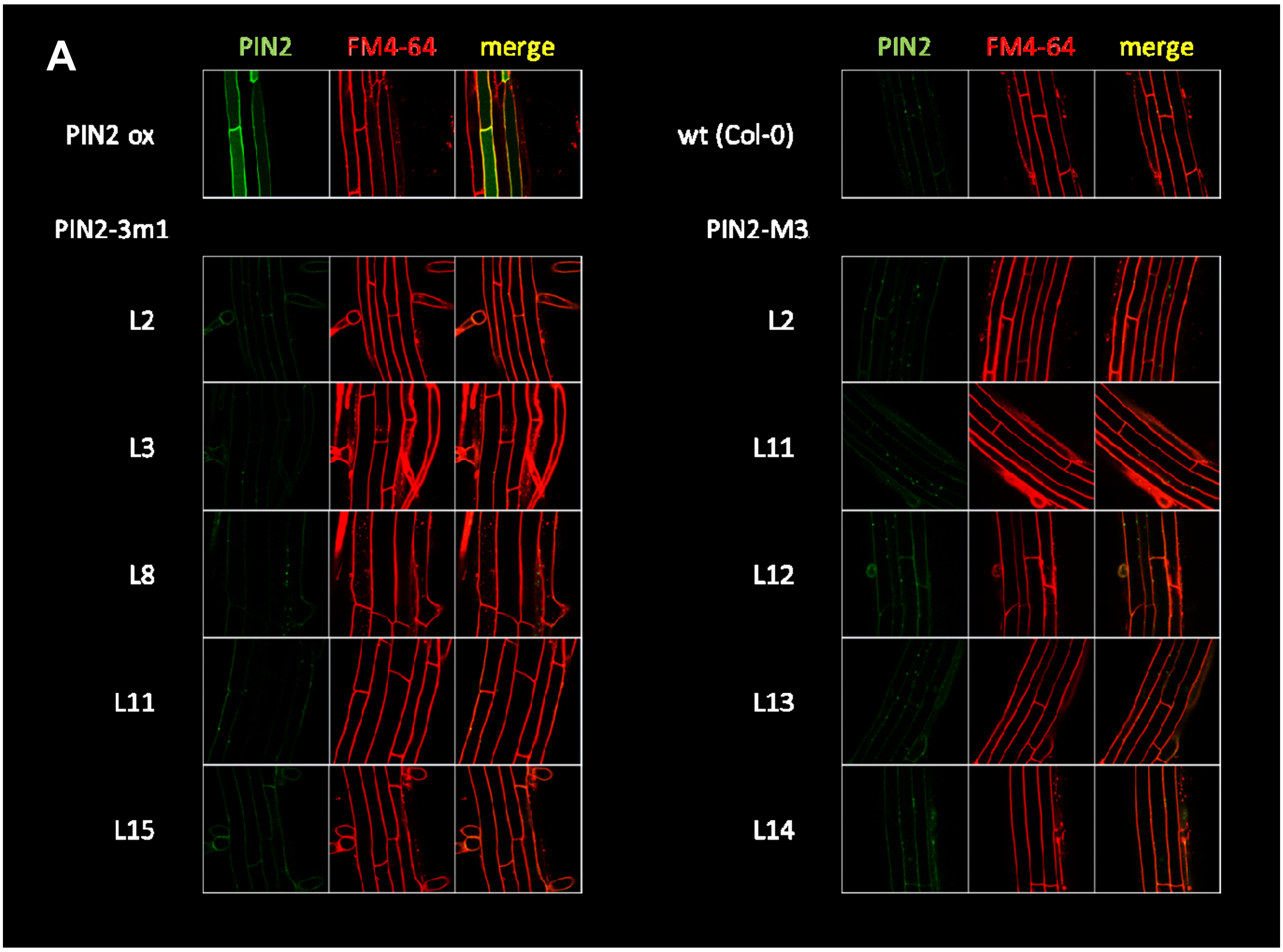

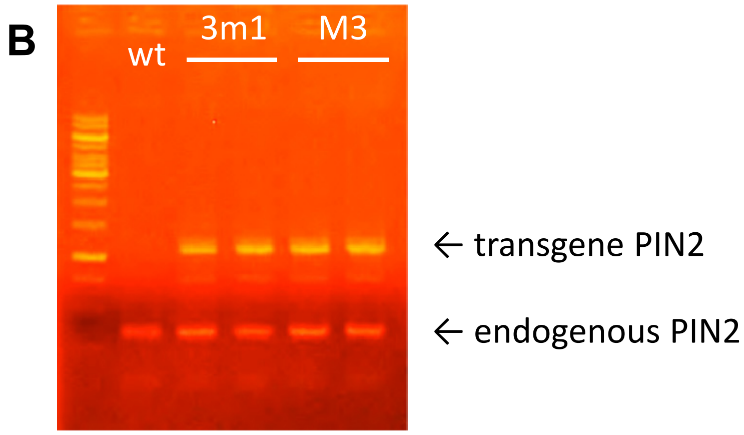

Figure S6. Confocal images and expression analysis of M3 phosphorylation-defective PIN2 lines. (A) Confocal images showing the subcellular localization of ProE7:PIN2, ProE7:3m1-PIN2 (PIN2-3m1A), and ProE7:M3-PIN2 (PIN2-M3A) in root hair cells. Four-day-old seedlings were used and stained with FM4-64 (2 µM). Five independent lines were observed for each mutant. **(B)** Expression analysis of GFP-fused PIN2 transgene by RT-PCR. Two independent lines were checked for each mutant.

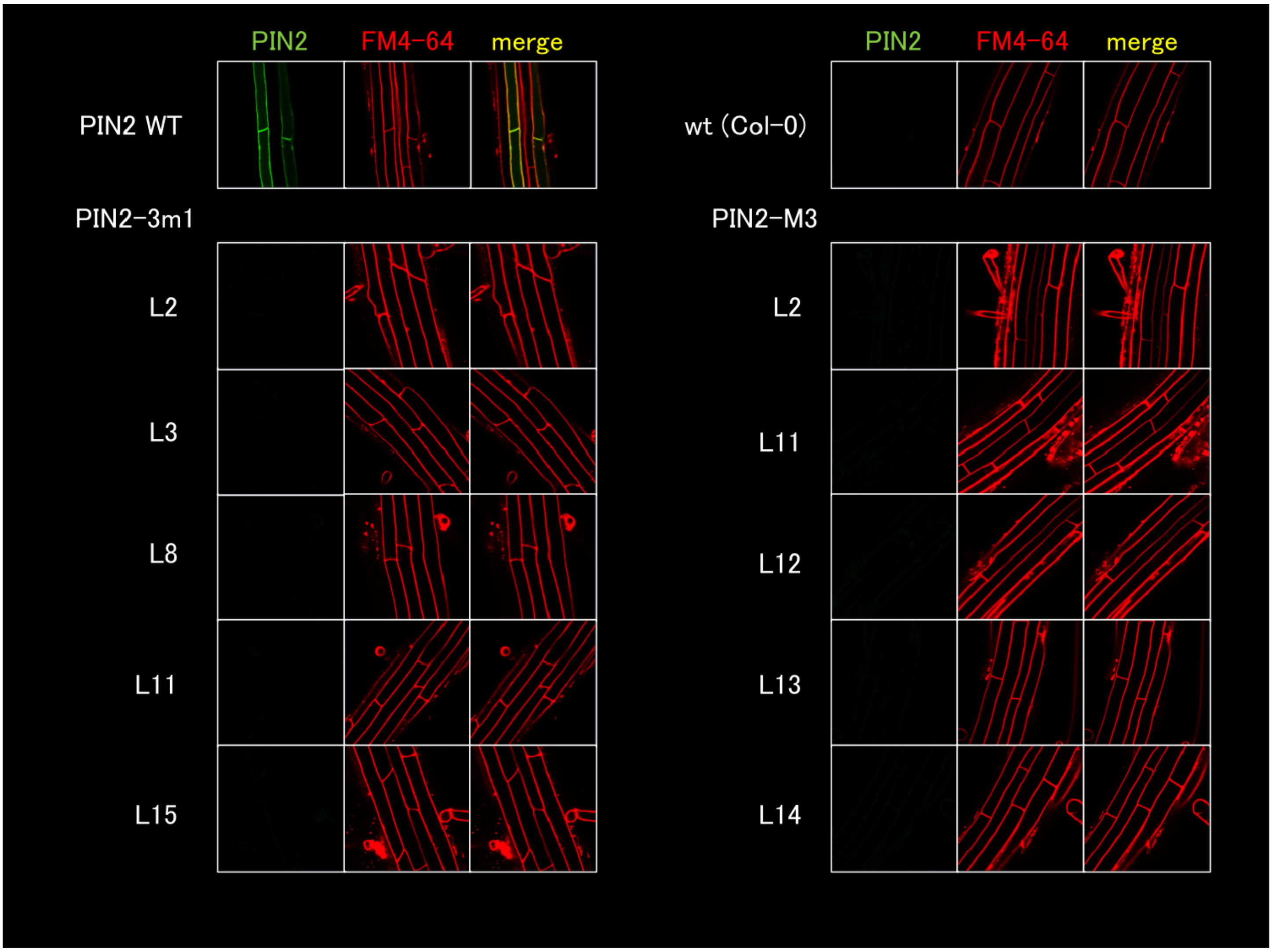

Figure S7. Confocal images of auxin-treated M3 phosphorylation-defective PIN2 lines. Confocal images showing the subcellular localization of ProE7:PIN2, ProE7:3m1-PIN2 (PIN2-3m1), and ProE7:M3-PIN2 (PIN2-M3) in root hair cells. Transgenic seedlings were treated with 5 μM NAA for 2 h and stained with FM4-64 (2 μM). Five independent lines were observed for each mutant.

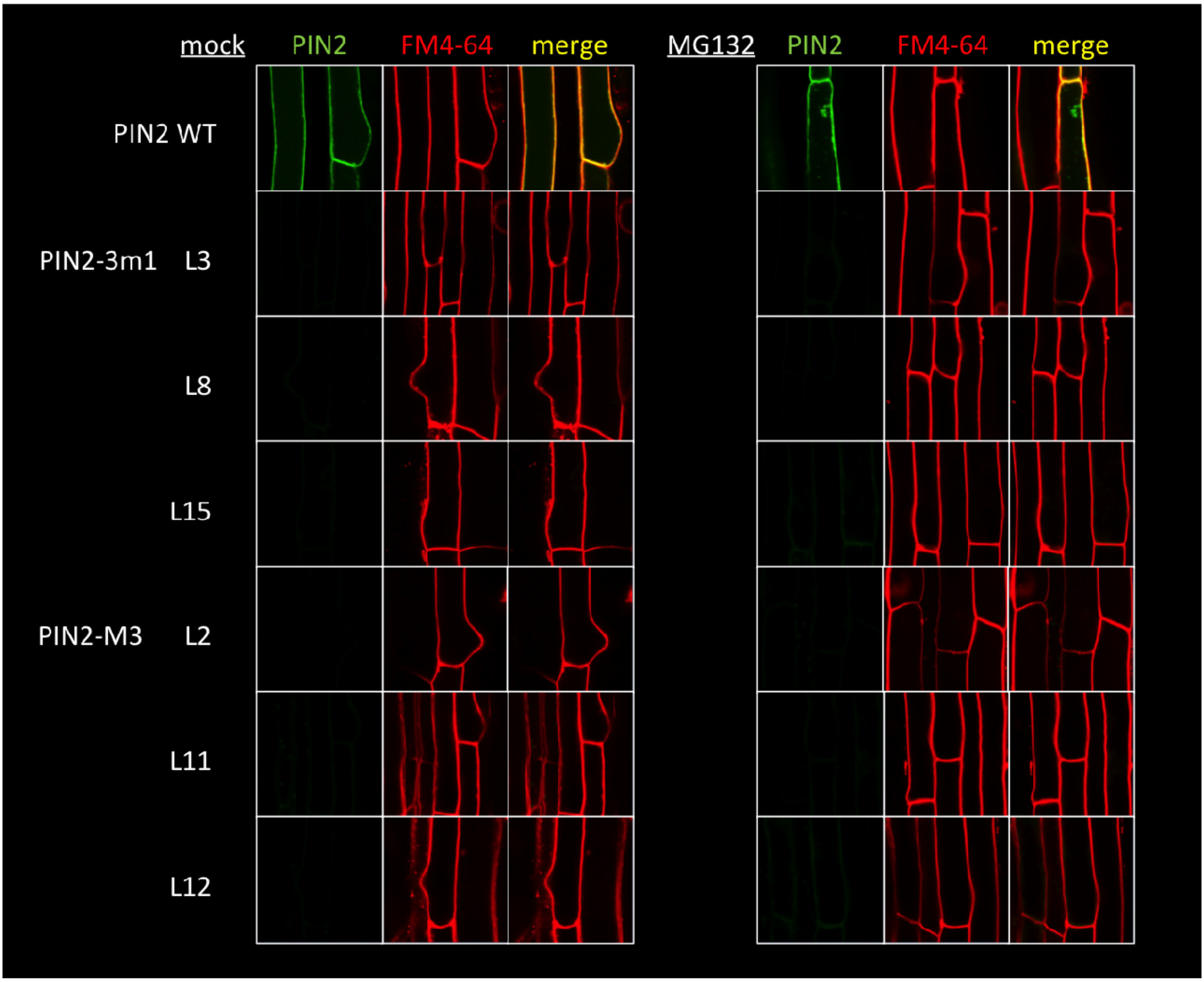

Figure S8. Confocal images of MG132-treated M3 phosphorylation-defective PIN2 lines. Confocal images showing the subcellular localization of ProE7:PIN2, ProE7:3m1-PIN2 (PIN2-3m1), and ProE7:M3-PIN2 (PIN2-M3) in root hair cells. Transgenic seedlings were treated with 25 µM MG132 for 2.5 h and stained with FM4-64 (2 µM). Three independent lines were observed for each mutant.

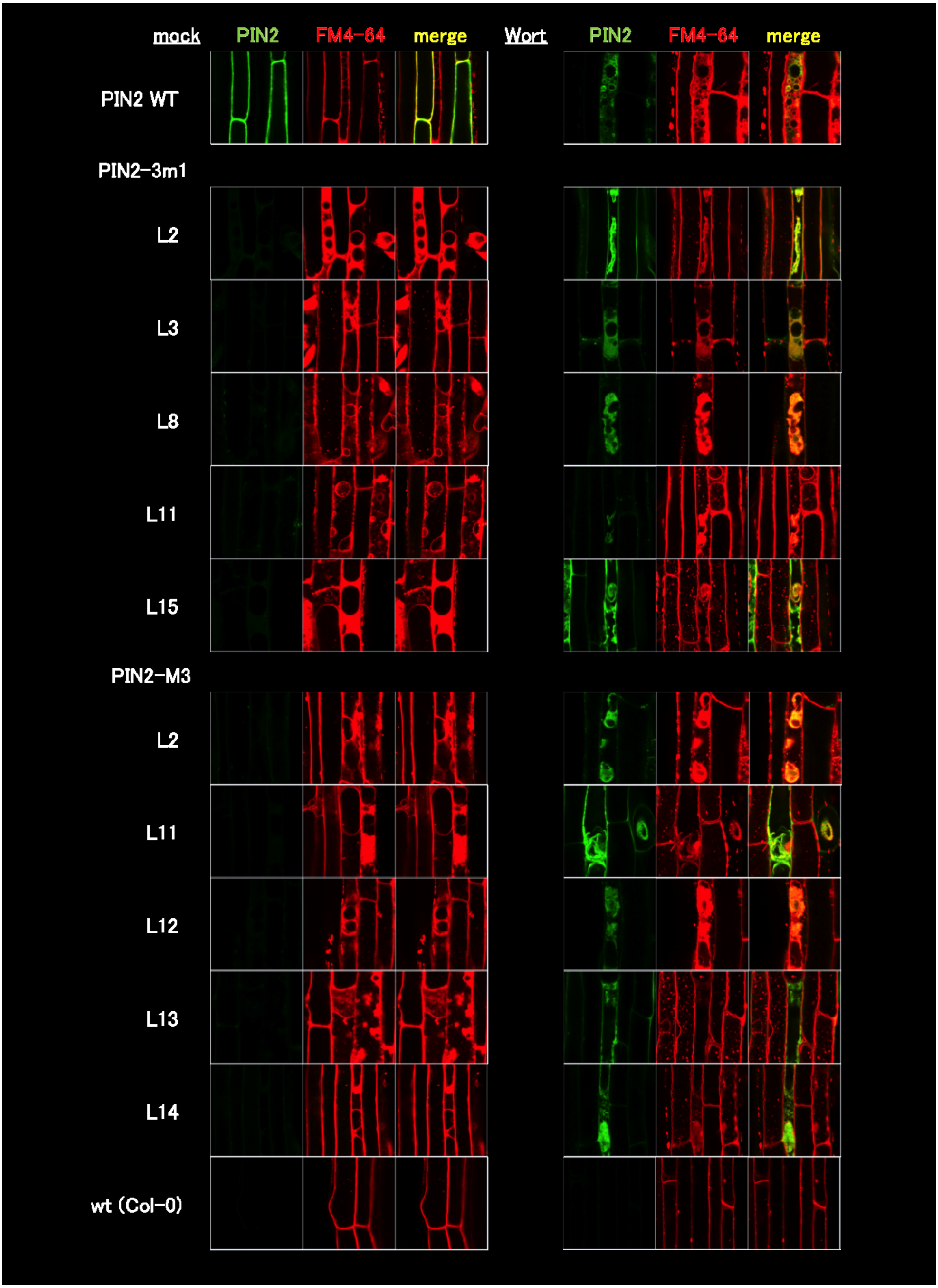

Figure S9. Confocal images of Wortmannin-treated M3 phosphorylation-defective PIN2 lines. Confocal images showing the subcellular localization of ProE7:PIN2, ProE7:3m1-PIN2 (PIN2-3m1), and ProE7:M3-PIN2 (PIN2-M3) in root hair cells. Transgenic seedlings were treated with 20 µM Wortmannin (Wort) for 12 h and stained with FM4-64 (2 µM, for 2 h). Five independent lines were observed for each mutant.

Table S1. Primer list

Subject	Template	Primer Name	Sequence (5' to 3')[1]	Subcloning Site
PIN1, 2, 7 mutation		E7 5'-tDNA	TGA AAA TCC CCT CTA TAT AAG ATT GTC TC	
PIN1, 7 mutation		mGFP4R118	CCG TAT GTT GCA TCA CCT TCA CCC T	
PIN2 mutation		GFP342RV	CTC GAC CAG GAT GGG CAC	
PIN1-3m1 mutation	ProE7:PIN1:GFP	PIN1 M3A-R1	TTG AGC CCT TCT CGA GTA AAT ATC AGA CCT TGC AGC ATT AGC ACG ACG AAC	SalI, NcoI
PIN1-M3 mutation	ProE7:PIN1-3m1:GFP	PIN1 M3A-R2	GGT TAG ATT CGC AGG TCT AGG TGC CGC AGA TAA	SalI, NcoI
PIN2-3m1 mutation	ProE7:PIN2:GFP	PIN2 M3A-R1	GTG AGC TTT GTT GAA TGA AGA GAT CAT TGA TGA GGC GGC ACT TGC TCT TCG AAC	SalI, XmaI
PIN2-M3 mutation	ProE7:PIN2-3m1:GFP	PIN2 M3A-R2	GGT GAG ATT TGC AGC TCG CGG CGC TAT CAT GGA	SalI, XmaI
PIN7-3m1 mutation	ProE7:PIN7:GFP	PIN7 M3A-R1	ACC ACC GTA AAA AGC TCT CCG AGC AGC GTT TGC TTT TCT CAC	SalI, NcoI
PIN7-M3 mutation	ProE7:PIN7-3m1:GFP	PIN7 M3A-R2	GGT GAG ATT AGC CGG ACG AGG AGC CAT ATT AGT	SalI, NcoI

[1] The mutation sites are underlined.
